# Accurate Monocular SLAM Initialization via Structural Line Tracking

**DOI:** 10.3390/s23249870

**Published:** 2023-12-16

**Authors:** Tianlun Gu, Jianwei Zhang, Yanli Liu

**Affiliations:** 1 College of Computer Science, Sichuan University, Chengdu 610065, China; zhangjianwei@scu.edu.cn (J.Z.); yanliliu@scu.edu.cn (Y.L.); 2 National Key Laboratory of Fundamental Science on Synthetic Vision, Sichuan University, Chengdu 610064, China

**Keywords:** monocular SLAM initialization, structural line tracking, geometric constraints, pose optimization

## Abstract

In this paper, we present a novel monocular simultaneous localization and mapping (SLAM) initialization algorithm that relies on structural features by tracking structural lines. This approach addresses the limitations of the traditional method, which can fail to account for a lack of features or their uneven distribution. Our proposed method utilizes a sliding window approach to guarantee the quality and stability of the initial pose estimation. We incorporate multiple geometric constraints, orthogonal dominant directions, and coplanar structural lines to construct an efficient pose optimization strategy. Experimental evaluations conducted on both the collected chessboard datasets and real scene datasets show that our approach provides superior results in terms of accuracy and real-time performance compared to the well-tuned baseline methods. Notably, our algorithm achieves these improvements while being computationally lightweight, without the need for matrix decomposition.

## 1. Introduction

Monocular simultaneous localization and mapping (SLAM) is a technique that involves tracking camera pose and constructing a sparse 3D map using a single RGB camera in real time. It serves as a fundamental technology in industries like augmented reality (AR) [1], autonomous driving [2], and indoor robotics. Despite its broad applications, the initialization process plays a pivotal role in monocular SLAM systems [3]. It has been a subject of special research focus in order to enhance system convergence and prevent localization failures.

However, the general monocular methods exhibit several limitations that can result in the degradation of initialization. Firstly, it necessitates a sufficient number of uniformly distributed feature matches between the initial frames. Secondly, a considerably large parallax is indispensable for the estimation of the initial pose. Lastly, and most significantly, it requires a longer convergence time or may even fail in man-made scenes that lack texture, such as those comprising plain floors and walls [4]. Consequently, attention has shifted towards structural-feature-based methods, which strive to fully exploit the geometric constraints present in man-made scenes.

In the context of the Manhattan World (MW) [5], the structural lines run parallel to one of the three mutually orthogonal dominant directions. The points where these parallel lines intersect in each dominant direction are commonly referred to as vanishing points (VPs) [6]. To be more specific, structural lines from various dominant directions may lie on the same structural plane, with their normals being parallel to one of the three dominant directions. On the other hand, tracking structural features poses more challenges compared to non-structural features due to their heightened sensitivity to noise. These structural features are involved in the pose optimization process as additional constraints subsequent to the initial pose estimation undertaken using unstructured features [7].

### Motivations and Contributions

Considering the inexorable occurrence of initialization errors in monocular SLAM systems caused by subpar initialization quality, we contend that it is more advantageous to minimize such errors to the greatest extent during the initialization phase, rather than depending on parameter adjustments in the stages of local optimization and backend optimization to attain consistent error smoothing across individual camera poses.

We propose a novel approach that utilizes structural features for camera initialization. This approach directly relies on structural features, from estimating the initial pose to computing 3D landmarks. The initialization optimization strategy is redesigned to overcome the problems associated with the vulnerability of non-structural feature initialization to factors such as texture and feature distribution. Due to the nature of structural features, they inherently contain more scene information. Merely using them as constraints for local optimization is evidently too narrow. We propose a creative approach by directly utilizing the structured features as the foundation for initialization, which greatly enhances the quality of initialization.

In this paper, we propose a novel initialization method for monocular SLAM that leverages structural features. In order to ensure the tracking quality of the structural features, we track structural line features across five consecutive frames using an enhanced line tracking algorithm. Three orthogonal dominant directions are extracted to cluster groups of structural lines, enabling the estimation of rotation by organizing vanishing points (VPs) [8]. Furthermore, we introduce vanishing point and plane optimization (VPPO), a technique that combines VPs and structural plane constraints to optimize the camera pose. The optimization results are superior when compared to non-structural features. We also design new metrics for evaluating the quality of the generated 3D structural feature maps. The main contributions of our work are as follows:

(1) A modified structural line feature tracking method that combines descriptor-based and optical-flow-based tracking. We aim to continuously track structural lines as much as possible in a sequence of image frames. Conventional line tracking methods suffer from problems of misalignment and missed matches, which pose challenges to the quantity and tracking stability of structural line matching. In particular, indiscriminate line matching introduces many non-structural lines, resulting in additional computational costs. By utilizing vanishing point constraints and performing line filtering in advance, we can reduce these costs and eliminate interference. Finally, we combine line descriptor matching and optical flow tracking to achieve the best matching results for structural lines.
(2) The introduction of VPPO as a novel camera pose optimization strategy that utilizes 3D–2D line reprojection and the geometric constraints of structural features. We extensively employ the geometric constraints of structural features to optimize the camera pose. Apart from reprojection error terms, we integrate error terms associated with geometric constraints into the optimization process. These encompass vanishing point direction error terms that effectively restrict camera rotation and plane error terms that positively influence camera translation.
(3) Novel evaluation metrics for assessing the map quality and evaluating the structural properties of the 3D line landmark. We propose two novel definitions for assessing the quality of initialization maps generated using structural features. This is crucial because the structural features within a scene are not solely tied to the pixels in the camera images. It is essential to evaluate and describe their spatial relationships within the generated initialization maps. Consequently, we introduce the evaluation of orthogonal and parallel positional relationships among the structural features in the initialization map as new indicators for assessing map quality. This also helps to further illustrate the advantages of our method in the generation of initialization maps. Instead of discrete 3D point coordinates, we now have 3D lines with geometric positional relationships.

## 2. Related Work

### 2.1. Classification of Visual Motion Estimation Methods

The methods for estimating camera motion from video can be categorized into three main groups: feature-based methods, direct methods, and semi-direct methods. Feature-based SLAM extracts features from images and uses feature descriptors to match and track features between frames. The camera motion is obtained through matrix decomposition, and the camera pose is determined by minimizing certain error terms (usually the reprojection error) [9]. While these methods demonstrate robust performance in highly textured environments, they are susceptible to failure when encountering repeated features that lead to feature matching errors. Moreover, the utilization of feature descriptors and matching features demands significant computational resources. The direct method achieves camera motion estimation by minimizing photometric errors, demonstrating good performance in weakly textured environments. It also exhibits some adaptability to motion blur, but is sensitive to changes in lighting conditions [10]. The semi-direct method combines feature extraction from the feature-based approach and feature tracking from the direct method. It utilizes photometric information for feature tracking instead of using descriptors, which helps to avoid errors caused by descriptor mismatches [11].

Feature-based methods often face challenges such as a decrease in the number of features, the uneven distribution of features, and motion blur caused by camera movement. These issues mainly stem from a reduction in correctly matched features. In contrast, direct methods, which do not rely on feature descriptors for matching, show better performance in cases involving repeated textures and rapid motion. Additionally, direct methods require fewer computational resources and offer faster processing speeds compared to feature-based methods [12].

### 2.2. Geometric Constraint Methods

In the early stages of research, the initial pose is primarily obtained through matrix decomposition [13]. PTAM [14] utilizes a five-point method [15] for fundamental matrix decomposition; this allows the pose estimation in real time. ORB-SLAM [16] and SVO [11] estimate both the fundamental matrix and homography matrix simultaneously during initialization, and then select the best model through multiple rigorous evaluations, which improves the accuracy of initialization. Compared to the fundamental matrix, planar homography imposes stronger geometric constraints and serves as a priori in numerous SLAM systems [17,18,19]. Additionally, the presence of line features in the scene is leveraged to compensate for the limited number of point features in environments. The improved semi-direct method PL-SVO [20] introduces line features as additional constraints in order to enhance performance.

Moreover, RGB-D methods, such as LPVO [21,22], utilize the Manhattan hypothesis to obtain the initial rotation from the structural planes, which can be directly computed in the depth image and significantly improves the accuracy of the rotation matrix. But in monocular SLAM systems, it is challenging to extract structural features during the initial stage. Therefore, structural features are often employed as optimization constraints after matrix decomposition-based initialization. The researchers in [23,24,25] extract structural line groups in three dominant directions and calculate the corresponding VPs for rotation optimization. Li et al. [26] utilize an encoder–decoder network to predict plane normals for extracting the structural planes and optimizing rotation.

### 2.3. Monocular Initialization Algorithms

The general initialization process of a monocular SLAM system begins with feature matching between frames to obtain the initial pose through fundamental matrix decomposition [27,28,29], and creates an initial 3D map through triangulation [30]. Subsequently, the system utilizes the PnP algorithm [31] to achieve 3D–2D matching and estimate the pose of the subsequent frames. The initialization process can be considered a form of Structure from Motion (SfM) [32,33], but it is more lightweight in terms of real-time performance by extracting sparse features instead of dense pixels.

Many methods also expand on the computation of the initial pose for scenes involving planes. This is necessary because the planar homography contains additional geometric information, which aids in reducing the uncertainty in determining the depth of pixel projection based on epipolar geometry [11,16]. In [34], an automatic real-time initialization module is implemented based on heuristic algorithms, which utilizes epipolar and homography geometry for the stable initialization of non-planar and planar scenes. All types of frameworks require reliable initialization to ensure optimal system performance [35], but as far as we know, there are no monocular initialization algorithms using structural constraints. We are the first to establish such a pipeline and use it in the man-made scene.

## 3. Algorithm Overview

**Figure 1:**
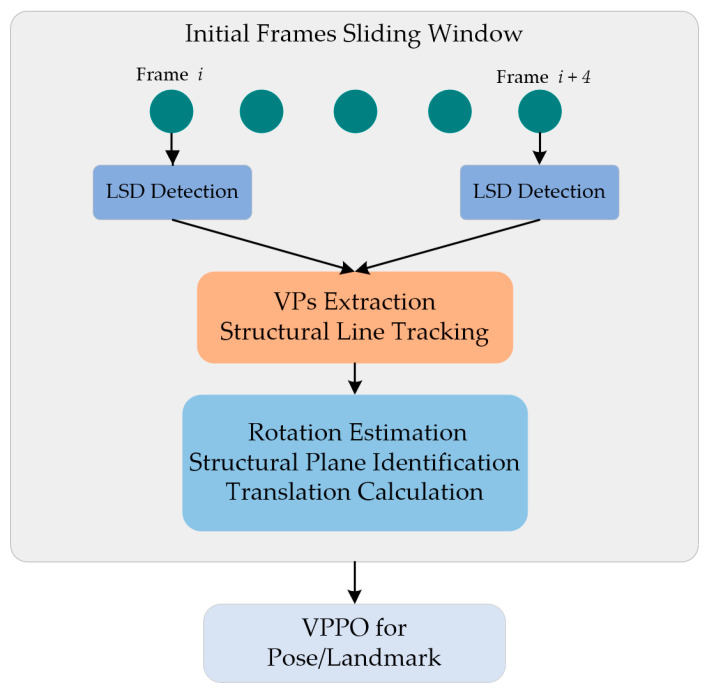
Structure of the proposed framework.

The workflow of the proposed initialization algorithm is depicted in Figure 1 and comprises several stages. In a sliding window five frames in length, we performed line detection using the line segment detector (LSD) [36], the extraction of vanishing points (VPs), structural line tracking, rotation estimation, the identification of structural planes, and the calculation of translation. Finally, utilizing the computed results from the sliding window, we proceeded with pose optimization and map initialization.

### Notations

We used the general pinhole camera model. The camera’s intrinsic matrix is *K*, which was calibrated offline, *O_w_* and *O_c_* represent the world and camera coordinate system, respectively, and the rotation and translation from *O_w_* to *O_c_* is indicated as *R_cw_* and *t_cw_*.

A projection relationship exists between the 3D point *X_w_* and its corresponding pixel *p_c_* in the image:

(1) $$p_{c}=\varphi \left(K\left(R_{cw}X_{w}+t_{cw}\right)\right)$$

the function *φ*(·) is defined to normalize the projected coordinate to the image plane, which is *φ*([*x*, *y*, *z*]*^T^*) = [*x*/*z*, *y*/*z*, 1]*^T^*.

The space plane Π is defined as (*n*, *d*), *n* is the normal vector, *d* is the distance from the plane to the origin, and 3D feature *X* on the plane satisfies the constraint |*n^T^X*| = *d*. If frames *c*_1_ and *c*_2_ observe plane Π at the same time, there is a transformation relationship between the matched pixels:

(2) $$H=K\left(R_{c_{1}c_{2}}+\frac{t_{c_{1}c_{2}}n_{c_{2}}^{T}}{d_{c_{2}}}\right)K^{-1}$$

H is homogeneous transformation, which can omit the *z* parameter involved in *φ*(·).

## 4. Structural-Line-Based SLAM Initialization

In this section, vanishing point extraction and structural line tracking will first be elaborated as the foundation of the proposed algorithm. Subsequently, the estimation of rotation, identification of structural planes, and calculation of translation will be discussed more specifically. Finally, after optimizing the camera pose with the VPPO optimization strategy, the construction of the initialization map will be accomplished. Non-structural lines filtered out by the vanishing point direction will not participate in tracking or pose computation and optimization, nor are they included in the scope of initialization map reconstruction.

### 4.1. VP Extraction and Structural Line Tracking

**Figure 2:**
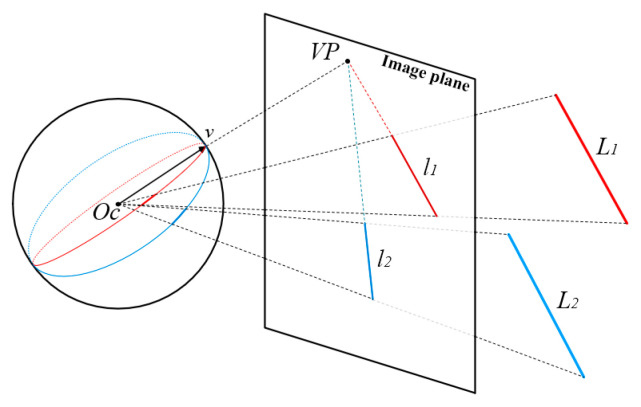
VP extraction via unit Gaussian sphere.

By mapping the lines on the image to a unit Gaussian sphere [37,38], the arcs formed by the parallel lines intersect at a common point. The direction from the sphere center to the intersection point represents the VP direction of the parallel lines. By clustering the intersections, the VPs and parallel lines of the image can be obtained, as shown in Figure 2. We adopted a coarse-to-fine strategy. Initially, we employed Gaussian sphere mapping to extract the VPs approximately and group the lines with the same direction. We then used orthogonal constraints to filter out the VPs and lines in three orthogonal directions. Finally, the RANSAC [39] method was applied to accurately solve the VP coordinates for each line group. We referred to the lines in each VP direction as structural lines, and tracked these structural lines between consecutive frames.

**Figure 3:**
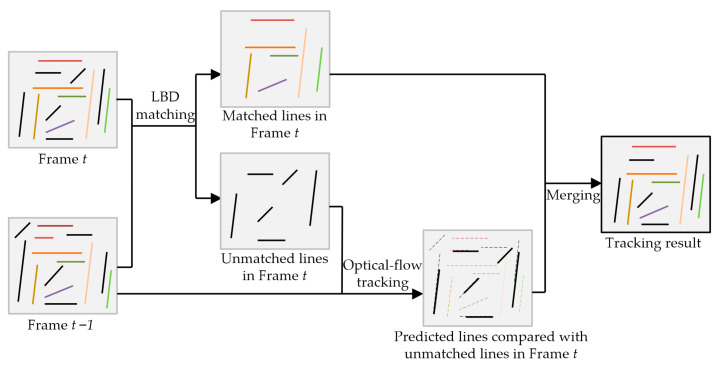
The modified line tracking strategy combining the line-based descriptor and pixel-based optical flow tracking. For ease of visualization, we placed frame *t* − 1 below frame *t*.

We utilized line matching techniques for the purpose of structural line tracking. While current line detection algorithms demonstrate satisfactory efficiency and stability, there is still potential for improvement in line tracking between frames. Line matching algorithms are frequently employed in SLAM systems [40,41,42], yet they often exhibit suboptimal continuous matching rates due to sensitivity to noise. To tackle this challenge, we propose a modified line feature tracking method based on line binary descriptor (LBD) [43] matching. This method aims to improve match probability by identifying additional matches among the unmatched lines, which should have been matched. Figure 3 demonstrates our approach that combines LBD matching with pixel-based optical flow tracking [44]. To handle unmatched lines, we sampled pixels along these lines and employed optical flow tracking in the subsequent frame. By calculating the pixel and angle distance between the unmatched lines, we could identify new matches. This methodology enabled us to reliably track line features across multiple frames.

### 4.2. Rotation Estimation with VPs

In our previous work [45], we proposed and validated a camera pose calibration algorithm based on VPs. In this study, we continue to utilize VPs to estimate camera rotation *R_cw_*:

(3) $$R_{cw}=\left[\frac{v_{x}}{{\left\|v_{x}\right\|}^{2}},\frac{v_{y}}{{\left\|v_{y}\right\|}^{2}},\frac{v_{z}}{{\left\|v_{z}\right\|}^{2}}\right]$$

where *v_x_*, *v_y_*, and *v_z_* are the VPs in camera coordinate *O_c_*.

### 4.3. Structural Plane Identification

**Figure 4:**
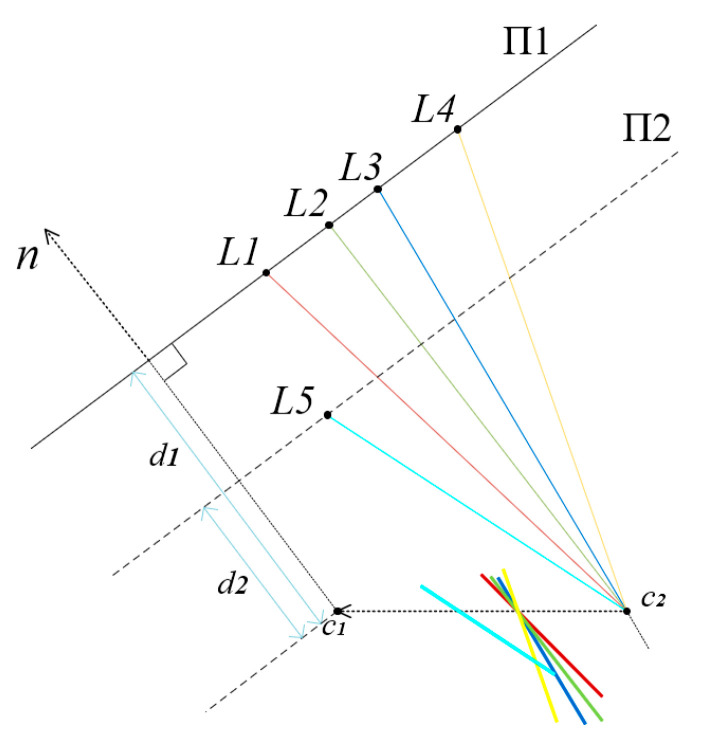
*c*_1_ and *c*_2_ are the camera centers of two initial frames on the baseline. The 3D lines *L*1, *L*2, *L*3, and *L*4 (orthogonal to the page) are parallel and coplanar on plane Π1, and their associated planes all intersect at a characteristic line (orthogonal to the page) [46], while *L*5 lies on another palne, Π2. *n* is the plane normal; *d*_1_ and *d*_2_ are the plane distances.

We extracted the structural plane based on the “characteristic line” (CL) [46], an invariant representation used to identify the coplanar lines from the parallel line set. Specifically, when a set of 3D lines are parallel and lie on the same plane, their corresponding 2D lines exhibit a shared CL, as shown in Figure 4. By clustering distinct CLs, we can derive the set of coplanar 2D lines {*C*} from the initial structural line set {*S*}. The plane on which {*C*} lies is referred to as the structural plane. Since the structural lines are parallel to the three principal directions, so are the normals of the structural planes.

### 4.4. Translation Calculation

**Figure 5:**
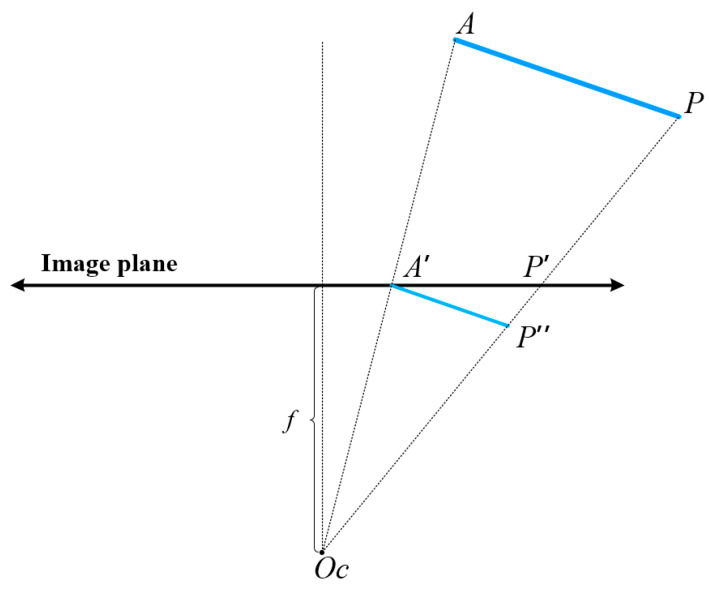
*A*′*P*′ is the x-axis line direction on the image plane, and its 3D line is *AP*. The world coordinate system is created with A as the origin *Ow*, and then *OcA* is the corresponding translation vector. Let *A*′*P*″ be parallel to *AP* and intersect *OcP* at *P*″, the translation *t_cw_* can be calculated from the similarity of the triangle *OcA*′*P*″ and *OcAP* with the known scale factor α in Equation (4), that α = $\left\|AP\right\|/\left\|A^{\prime }P^{\prime \prime }\right\|$.

We established the world coordinate system on the structural plane that contained the largest number of coplanar lines. A structural plane typically includes structural lines aligned with two dominant directions, which are defined as the x and y directions of the coordinate system. A structural line was selected from both the x and y directions, respectively, as the x-axis and y-axis, and the intersection of these two lines was taken as the origin of the coordinate system. The z-direction can be obtained by taking the cross product of the x and y directions. Based on stable structural line tracking, we could easily calculate the translation of the initialization frame [45], as shown in Figure 5. *A*′*P*′ is projected from *AP*, *P*″ the intersection of the line (*O_c_P*) with the line *L* passing through *A*′ and whose direction is *A*→*P*. It is assumed the length of *AP* is known, based on our experience, set it to be 1000 units in length. Otherwise, *t_cw_* will be calculated up to a scale factor *α*. As *O_c_A*′*P*″ and *O_c_AP* are similar triangles, the lengths of *O_c_A*′ and *O_c_P*′ can be directly calculated from pixel coordinates and camera intrinsic parameters. *AP* is one of the axes of the world coordinate system, rotating its direction through the *R_cw_* to obtain the direction of *A*′*P*″ in the camera coordinate system. Moreover, *A*′*P*″ intersects *O_c_P*′ at point *P*″, obtaining the length of *A*′*P*″. So, we obtain the translation vector *t_cw_*:

(4) $$t_{cw}=O_{c}A=O_{c}A^{\prime }\cdot \frac{\left\|AP\right\|}{\left\|A^{\prime }P^{\prime \prime }\right\|}$$

.

### 4.5. Map Initialization and Optimization

Under the plane constraint *n^T^X* = *d*, it should be noted that the normal vector *n* here is away from the center of the camera; therefore, the projection of points on the 3D plane in the direction of *n* is equal to ‘*d*’ rather than ‘−*d*’. We rewrite the camera projection Equation (1) into a form that includes the depth value *z*:

(5) $$zK^{-1}p_{c}=X_{c}$$

Among the plane parameters (*n*, *d*), *n* is one of the VP directions, and *d* can be calculated from the projection of *t_cw_* in the *n* direction, because the *O_w_* is directly established on the structural plane where the coplanar structural lines are located. Combining the plane constraint *n^T^X_c_* = *d* in *O_c_* and Equation (5), the depth *z* can be calculated as:

(6) $$z=\frac{d}{n^{T}K^{-1}p_{c}}$$

We can utilize the aforementioned method to compute the depth of each feature in all frames and subsequently calculate the average depth for a single landmark. In particular, we have designed an optimization strategy based on vanishing points and plane constraints (VPPO) to further optimize the camera pose and landmarks.

(7) $$\arg\min\left(\sum \left\|e_{p}\right\|+\sum \left\|e_{l}\right\|+\sum \left\|e_{o}\right\|+\sum \left\|e_{\pi }\right\|\right)$$

where the line endpoint reprojection error term is:

(8) $$e_{p}=p_{c}-\varphi \left(K\left(R_{cw}X_{w}+t_{cw}\right)\right)$$

The error term with respect to the line direction *v_L_* is:

(9) $$e_{l}=d_{l}\cdot \varphi \left(K\left(R_{cw}v_{L}+t_{cw}\right)\right)$$

The orthogonal error term with respect to the line direction *v_L_* and plane normal vector *n* is:

(10) $$e_{o}=R_{cw}v_{L}\cdot n$$

The coplanar error term is:

(11) $$e_{\pi }=\left(R_{cw}X_{w}+t_{cw}\right)\cdot n-d$$

## 5. Experimental Results

In this section, we test the effectiveness of the proposed algorithm on our collected datasets and the open-source dataset ICL-NUM [47], which possesses structural features. First, the dataset and evaluation metrics are introduced, and the implementation details are briefly described. Then, several common feature-based initialization methods, including those based on epipolar geometry and plane-based methods, are proposed for comparison. Finally, our method is compared with the baselines for both accuracy and speed.

### 5.1. Dataset, Evaluation Metrics

**Figure 6:**
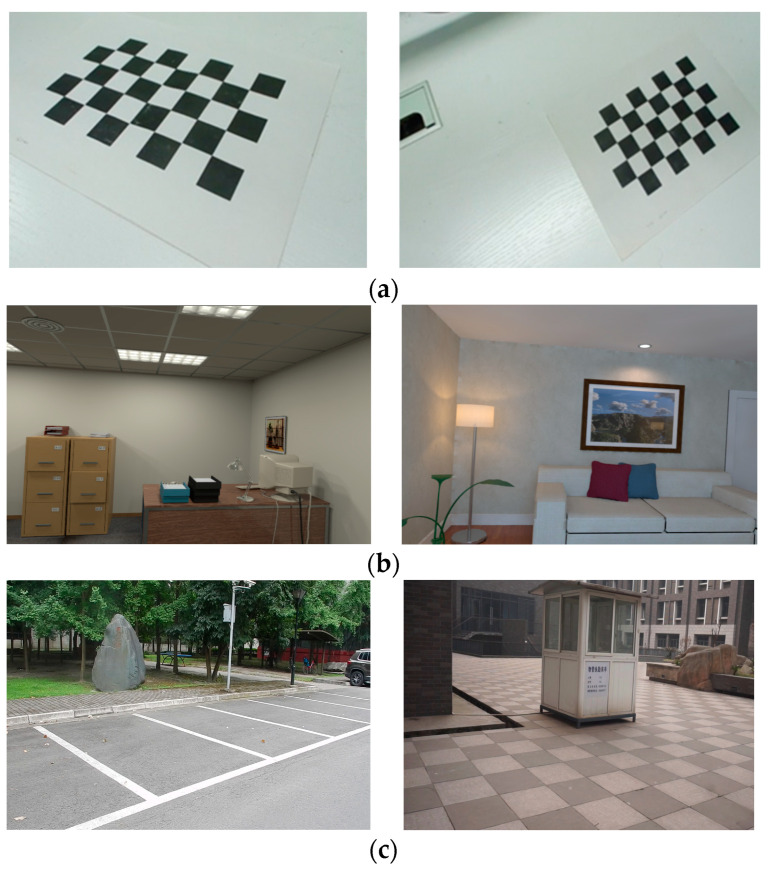
Samples of dataset images. (**a**) The sample images of self-collected chessboard datasets. (**b**) The sample images of open-source ICL-NUM datasets. (**c**) The sample images of the self-collected real-world scenes datasets.

We captured eight video sequences with different motion trajectories, speed, and perspectives. A chessboard was placed in each video, which allowed us to obtain the ground truth from the known grids. Some of the chessboard images are shown in Figure 6a. The sample images of open-source ICL-NUM datasets are shown in Figure 6b. Sample images of real-world scenes collected by us are represented in Figure 6c. For the chessboard datasets, we can acquire the accurate trajectory of the camera along with the plane parameters by utilizing the PnP algorithm, which relies on the known chessboard rig. For ICL-NUM datasets, the ground truth has already been provided. For the self-collected datasets of the real-world scenes, we obtain the camera trajectory through COLMAP [48]. Table 1 presents some parameters of the experimental datasets. Table 1 presents some parameters of the image sequences from each dataset involved in the experiment.

The evaluation metrics we used were as follows: the absolute translation error (ATE), to measure the quality of camera pose, the plane distance error (PDE), and the root-mean-square of reprojection error (RMSE) for map quality. In order to measure the map quality of the structured scenes, we specially designed two evaluation metrics to estimate the positional relationship between 3D structural landmarks: the orthogonal angle error (OAE) and the parallel angle error (PAE). Structural lines exhibit specific geometric positional relationships. Lines originating from distinct vanishing points are perpendicular to each other, while lines stemming from the same vanishing point are parallel. During the initial map generation process, it is crucial not only to minimize the average reprojection error of the 3D landmarks, but also to ensure the precision of their relative geometric positions, particularly for those with specific geometric relationships. Orthogonal angle error (OAE) is employed to estimate the angular error between 3D lines in different vanishing point directions, while parallel angle error (PAE) estimates the angular error between 3D lines in the same vanishing point direction. This is imperative, as it aids the system in selectively choosing high-quality 3D landmarks for optimization during the back-end optimization phase.

We tested all baseline methods on each image sequence with a maximum frame count of 60 for each test. The average error was computed with a length interval of 10. We conducted multiple experiments on consecutive frames from each image sequence. The number of image sequences, test repetitions per sequence, and the number of frames involved in the experiments are presented in Table 1.

### 5.2. Implementation Details

The proposed method was implemented using OpenCV [49] and the Google Ceres-Solver toolkit [50] on a PC with a Core i7-9750H CPU (2.60 GHz, six cores, 32 GB memory). The image resolution used in the calculation process is 640 × 360, and there is no GPU acceleration in the whole process. The processing time of a single frame image is less than 0.03 s, which makes this algorithm have a frame rate of at least 33 frames per second without any software and hardware acceleration.

### 5.3. Baseline Methods Discussion

We selected typical monocular initialization algorithms to compare with our proposed algorithm as far as we could. We grouped the monocular initialization methods into the two categories outlined below.

#### 5.3.1. Aggregation-Based PnP

We chose two aggregation-based methods based on the two-frame matrix decomposition. The first method is PnP from PTAM [14] based on epipolar geometry. The fundamental matrix is decomposed from two views, and then the landmarks can be obtained by triangulation. It applies PnP to estimate the poses of the remaining views and uses triangulation to progressively obtain landmarks. This method is easily affected by many factors, such as the movement between the two initialized views being too small, or the matching features being too few or unevenly distributed.

The second one is Plane-PnP based on plane constraints from ORBSLAM [16]. The homography matrix satisfied by the coplanar feature can be roughly estimated by RANSAC, and decomposing the homography matrix will obtain the initial pose. Triangulation is employed during the initial mapping stage. Subsequently, utilizing the initialized map, PnP is applied to accomplish 3D-2D pose estimation for subsequent frames. Likewise, triangulation is utilized to achieve incremental updates of the map. This method is currently the first choice for monocular SLAM initialization.

#### 5.3.2. Multi-Frame Optimization

We chose different strategies to optimize the initial pose. To further enhance the quality of the obtained pose and landmarks during the initialization process, bundle adjustment (BA) and plane bundle adjustment (PBA) are employed across multiple frames. For BA, the optimized variables include poses and landmarks, while PBA adds plane constraints, requiring the landmarks participating in the optimization to be coplanar in 3D space.

### 5.4. Ablation Study for Line Tracking

**Figure 7:**
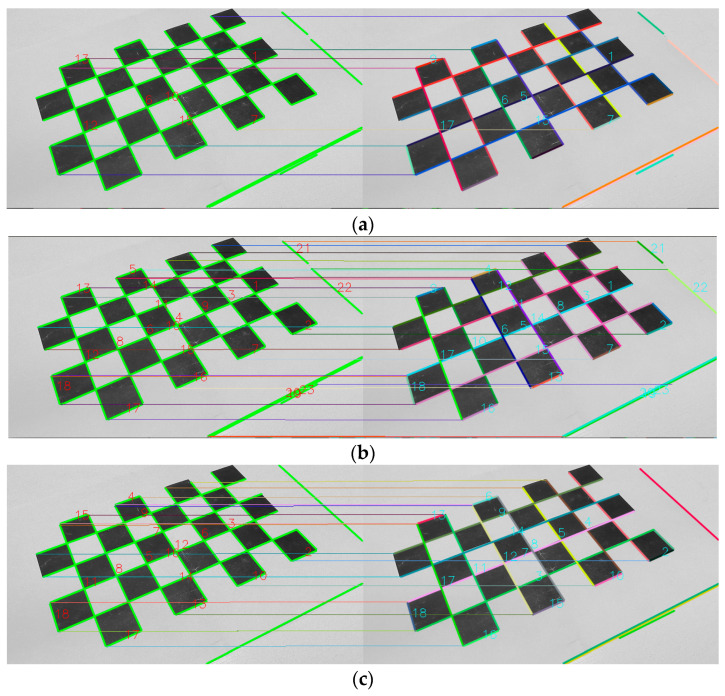
Line tracking: (**a**) only 7 lines matched with LBD at the rough matching step because the textures are monotonous and repetitive; (**b**) the number of matches increases up to 20 after enhanced matching; (**c**) line tracking after a five-frame interval, but still maintaining high-quality matching with 16 matches.

We conducted an ablation study for line tracing, showing the necessity for an improved line tracing strategy between consecutive frames. We visualized the results of the descriptor-based line matching between two frames, and the improved line matching with the combination of LBD matching and pixel-based optical flow tracking, as shown in Figure 7. There is a significant difference in the number of line matches between the two. The strategy before the improvement is obviously unable to meet the needs of stable and continuous structural line tracking between multiple consecutive frames. The modified method can even obtain ideal transitive matching results in lengths of more than five consecutive frames.

### 5.5. Comparison with Aggregation-Based PnP Methods

**Figure 8:**
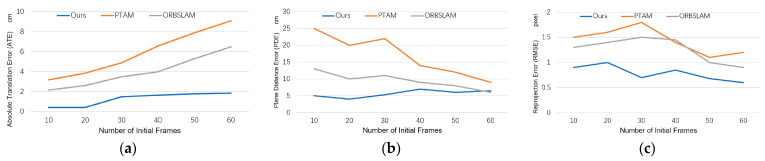
ATE, PDE, and RMSE of our method and aggregation-based baselines.

We compared our method with two PnP methods. In Figure 8, it can be seen that our proposed method is superior to the PnP and Plane + PnP algorithms in terms of average ATE, PDE, and RMSE performance after testing all datasets. Especially in ATE, our method effectively avoids the accumulation of translation errors, while other methods continue to accumulate translation errors. In terms of PDE and RMSE, all methods decrease the cumulative errors as the initialization frame increases and more multiple perspectives features are added, but our method still has higher accuracy. PnP is relatively less stable, but, like other methods, more initial frames will bring better results.

### 5.6. Comparison with Multi-Frame Optimization Methods

**Figure 9:**
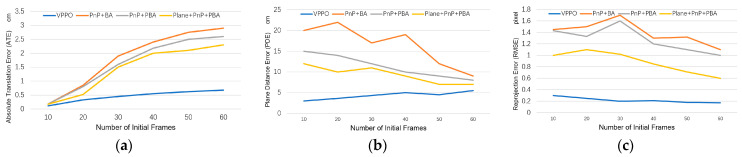
ATE, PDE, and RMSE of our method and the optimization baselines.

We compared our optimization method, VPPO, with different optimization methods. The multi-frame optimization strategy allows us to fuse as much information from multiple frames as possible, so that the final result has the lowest cumulative error and the highest positioning accuracy. We combined different initial pose algorithms with different optimization strategies and compared them with our proposed method. As shown in Figure 9, VPPO presents a huge accuracy advantage. It achieves significantly better pose accuracy than BA and PBA from the very beginning frames and performs the best among all methods when using more than 30 frames. Our method is also fast and accurate in estimating plane positions, which also results in a relatively minimal RMSE.

BA optimization can, indeed, effectively improve the quality of initialization, especially after adding plane constraints. For PnP + BA, the increase in multi-frame information will bring about greater optimization fluctuations, because there is no filtering for low-quality road signs. With the addition of plane constraints, the results of PnP + PBA are significantly improved. Plane + PnP + PBA obtained the initial pose based on coplanar features from the beginning, and then optimized with PBA achieves the second-best performance.

We also found that with optimization based on BA or PBA, as the amount of information increases, the error will first increase and then decrease, because the quality of each pose cannot be guaranteed, especially the poses in the first few frames.

We counted the running time of different methods, including the pose estimation and optimization process. As shown in Table 2, the results of each method tend to be stable at 50 frames, and we counted the average time consumed by each method in this process. Our method runs fastest among the experimental methods for both the pose estimation time and the optimization time. Because the number of features involved in each frame in our method is the smallest, the number of iterations of RANSAC is much smaller than for other methods. In addition, our method does not involve matrix decomposition.

### 5.7. Initialize Map Quality Evaluation

We specifically designed an accuracy assessment for the positional relationships between landmarks to test whether the generated initial map conforms to the expected structural assumptions. Based on the initial poses obtained from PnP and Plane + PnP, the line features are triangulated. Similar to our method, the grouping of structural lines is obtained using VP direction constraints, and, subsequently, the OAE and PAE are calculated separately with 50 initialization frames, as presented in Table 3.

We find that our method preserves the best relative relationships between landmarks in the initialized map for structural scenes. In contrast, other methods without incorporating structural optimization constraints result in uncorrelated landmarks in the initialized map, rendering the obtained OAE and PAE values meaningless for evaluation.

### 5.8. Qualitative Analysis

**Figure 10:**
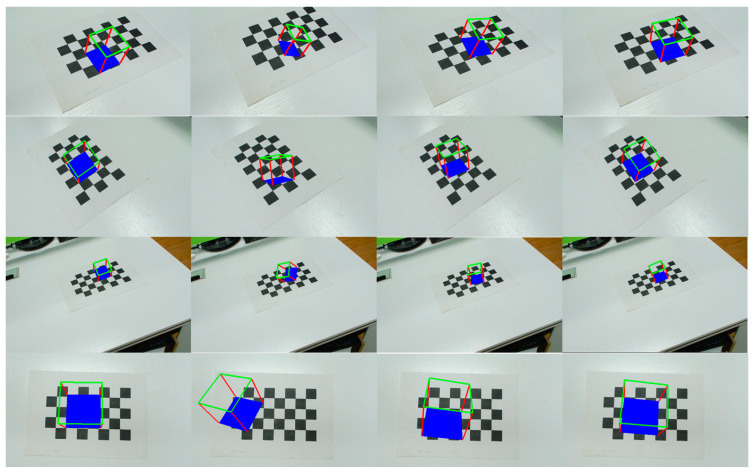
Initial pose visualization on chessboard images. The cube with the blue bottom draws on the plane extracted from the landmarks. From left to right are the results of VPPO, PnP + BA, PnP + PBA, and Plane + PnP + PBA.

Figure 10 demonstrates the qualitative results of all tested methods using the chessboard datasets. Our work, VPPO, shown in the first column, obtained the best results: the blue bottom lies exactly on the plane, and its orientation is exactly aligned with the chessboard. The second column, PnP + BA, yields the worst performance because there is no plane constraint involved. Although PnP + PBA in the third column uses coplanar landmarks during optimization, its results are still inferior to Plane + PnP + PBA in the fourth column due to the error of triangulation.

**Figure 11:**
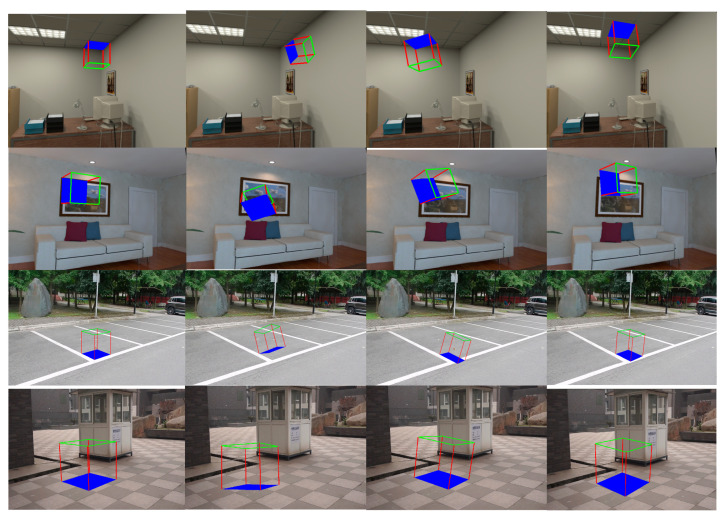
Initial pose visualization in real scene images. From left to right are results of VPPO, PnP + BA, PnP + PBA, and Plane + PnP + PBA.

We also validated our method using real scenes with structural features. As shown in Figure 11, the first and second rows depict indoor scenes, where the structural features mainly come from the walls, such as the ceiling texture and the picture frame on the wall. The third and fourth rows depict outdoor scenes, where the structural features mainly exist on the ground, such as parking lot boundaries and the seams of marble floor tiles. Our method directly constructs a world coordinate system based on these structural lines on the walls, achieving the highest quality of pose accuracy.

Our method is not sensitive to the number of structural lines in the scene. It is evident from our experiments that a minimum of three coplanar lines is required for localization, including two lines with the same direction and one from another orthogonal direction. The impact of the number of lines on tracking speed is not significant. However, it should be noted that we limited the number of lines involved in the optimization process to exclude 3D lines with large angle errors as much as possible.

## 6. Conclusions

In this paper, we present a novel initialization method for monocular SLAM based on structural features. Through the continuous tracking of structural lines, we extract vanishing points and structural planes to estimate camera poses without matrix decomposition and feature triangulation. We validate the performance of our method using our chessboard datasets. The experimental results demonstrate that the performance of the proposed method is better than the baseline methods both in terms of pose accuracy and map quality. Our method also has certain limitations. We require the presence of structural features in the scene, such as vanishing points and structural lines. However, in many cases, this requirement cannot be fulfilled, posing a challenge to the applicability of our method. Nevertheless, once successfully applied, our method can provide robust initialization results, which is highly attractive for SLAM in structural environments. Therefore, our future work will focus on integrating our method into existing SLAM systems. We aim to expand the monocular SLAM initialization module and make it one of the optional initialization strategies. Additionally, we will further enhance the backend optimization strategy based on structural lines to maximize the utilization of structural features in coordination with the frontend.

## Figures and Tables

**Table 1:** Experimental dataset parameters.

Datasets	Number of Sequences	Frames per Test	Repeated Test per Sequence	Frames Involved in Test per Sequence
Chessboard	8	60	6	360
ICL-NUM	2	60	10	600
Real-world scenes	2	60	10	600

**Table 2:** Average pose estimation and optimization time cost of different methods with 50 initialization frames.

	VPPO	PnP + BA	PnP + PBA	Plane + PnP + PBA
Pose estimation time (ms)	3.3	7.6	6.8	10.8
Optimization time (ms)	25.1	60.3	45.5	35.4

**Table 3:** Average OAE and PAE of different methods with 50 initialization frames.

	VPPO	PnP + BA	PnP + PBA	Plane + PnP + PBA
OAE (radian)	0.0352	0.1835	0.1785	0.1584
PAE (radian)	0.0385	0.3212	0.1973	0.1885

## Data Availability

Data are contained within the article.
